# Homeostatic proliferation of memory T cells and expansion of the HIV-1 latent reservoir

**DOI:** 10.1186/1758-2652-13-S3-O6

**Published:** 2010-11-04

**Authors:** A Bosque, V Planelles

**Affiliations:** 1 Department of Pathology, University of Utah, Salt Lake City, Utah, 84132, USA

## Background

Homeostatic proliferation is the ability of T cells to divide in the absence of activation. This process is mediated, preferentially, by the cytokine IL-7 and plays an important role in the long-term maintenance of immunological memory. Therefore, it is conceivable that the latent viral reservoir may be perpetuated or even expanded through homeostatic proliferation of memory T cells.

## Methods

We have developed a system whereby naïve cells from the peripheral blood of healthy donors are induced to undergo normal development *ex vivo* in the presence of the appropriate cytokine cocktails and antigenic stimulation through CD3/CD28. These cells are infected while in the activated state, and return to quiescence as central memory cells (T_CM_). Infection of these ex-vivo-generated memory cells leads to latency with a high frequency and results in the formation of a polyclonal population of integrated viruses. Using this paradigm, we have explored the influence of homeostatic proliferation of T_CM_ on HIV-1 latency.

## Results

We have examined the influence of cell cycle on viral reactivation entry and found that memory lymphocytes harbouring latent proviruses are perfectly capable of cell division in the absence of viral reactivation when incubated in the presence of IL-2+IL-7. On the other hand, we have also observed that a combination of IL-2 and IL-7 induces inefficient viral reactivation (20% to 30% of that which is observed with antiCD3/antiCD28). While IL-2+IL-7 treatment induces inefficient reactivation, this cytokine cocktail triggers vigorous cellular proliferation. Our *ex vivo* model demonstrates that under these conditions, the net effect of IL-2+IL-7 treatment is an expansion, not a contraction, of the latent reservoir. We also examined the signalling pathway that leads to HIV-1 reactivation upon IL-2+IL-7 incubation. Our preliminary results indicate that reactivation by IL-2+IL-7 is both NFAT and NFκB independent.

## Conclusions

Our results demonstrate that when latently infected NP cells are incubated with IL-7, cells proliferate in the absence of efficient HIV-1 reactivation. This concept has great relevance to therapy because of the implicit consequence that the latent reservoir may also be subject to homeostatic expansion. This mechanism could be contributing to the persistence of HIV-1 latency and should be taken into account when designing anti-latency treatments.

